# Fetal Environment and Glycosylation Status in Neonatal Cord Blood: A Comprehensive Mass Spectrometry-based Glycosylation Analysis: Erratum

**DOI:** 10.1097/01.md.0000483156.12046.71

**Published:** 2016-05-06

In the article “Fetal Environment and Glycosylation Status in Neonatal Cord Blood: A Comprehensive Mass Spectrometry-based Glycosylation Analysis”,^1^ which appeared in Volume 95, Issue 14 of *Medicine*, the locants in Figure 1 were incorrectly switched in the figure legend. The article has since been corrected online.
